# Burden of cataracts in developing countries: a trend analysis based on data from China, 1990 to 2021

**DOI:** 10.3389/fmed.2025.1550788

**Published:** 2025-04-29

**Authors:** Cancan Zhang, Pingping Li, Lurun Yu, Lu Li

**Affiliations:** Department of Eye Center, Renmin Hospital of Wuhan University, Wuhan, China

**Keywords:** cataracts, epidemiology, disability-adjusted life years, health burden, visual impairment

## Abstract

**Objective:**

To investigate the trends in cataract-related blindness and vision impairment burden among cataract patients in China, the largest developing country, from 1990 to 2021, providing evidence to inform future cataract prevention and control strategies in developing countries.

**Patients and methods:**

This study utilized data from the Global Burden of Disease (GBD) 2021 database to assess the prevalence, age-standardized prevalence rates (ASPR), and years lived with disability (YLDs) associated with cataract-related blindness and visual impairment in China and other regions from 1990 to 2021. Joinpoint regression, decomposition analysis, and ARIMA modeling were employed to analyze trends and predict future disease burden.

**Results:**

From 1990 to 2021, the number and crude rate for cataract related blindness and visual impairment increasing significantly in China. The age-standardizes rate increased from 878.30 to 989.58 per 100,000 population for cataract prevalence, and from 73.40 to 61.39 per 100,000 population for cataract YLDs. The majority of number of prevalence and YLDs are concentrated in the 65–89 age range. Women had higher cataract prevalence and YLDs than men. Population aging is the primary driver of the growing burden of cataract, contributing 73.12% and 98.3% to the increases in prevalence and YLDs, respectively. By 2035, the crude prevalence rate and crude YLDs rate of cataracts are projected to increase by 26.06% and 23.03%, respectively, compared to 2021. The age-standardized prevalence and YLDs rate attribute to cataract in China were lower than global averages and ranked third among neighboring countries.

**Conclusion:**

Despite advancements in cataract treatment that have alleviated some of the disease burden, population growth and aging continue to drive the increasing prevalence of cataract-related vision impairment in China. Addressing gender disparities and improving access to cataract surgery and preventive care are crucial for reducing this growing burden.

## Introduction

Cataracts are a degenerative disease of the lens, associated with multiple factors such as age, environment, and metabolism (1, 2). They lead to changes in refractive properties and increased light scattering, resulting in blurred vision, visual field defects, and even vision loss (3). Cataracts are one of the leading causes of blindness globally. In 2010, cataracts accounted for 33.4% (10.8 million cases) of global blindness and 18.4% (10.8 million cases) of moderate-to-severe visual impairment (MSVI) (4). It is well known that the epidemiological impact of cataract-related blindness varies across countries. In well-developed countries with good healthcare, cataracts account for only 5% of blindness, whereas in developing countries, cataracts still account for 50% of blindness (5, 6). With population growth and the ongoing impact of global aging, the proportion of blindness caused by cataracts is expected to continue rising. The impact of cataracts on vision loss, particularly among the elderly, exacerbates the risk of dementia, increases the likelihood of falls and road traffic accidents, significantly affects quality of life, and ultimately leads to higher mortality rates (7, 8). To date, surgery remains the only effective treatment for cataract patients (9). Due to advancements in modern cataract surgery techniques, the procedure is now considered relatively safe and can significantly improve visual function (10). Studies have shown that timely and equitable access to cataract surgery can prevent fall-related injuries and support healthy aging (11, 12).

Despite the remarkable efficacy of cataract surgery, global awareness of the procedure remains inconsistent (13, 14). Economic factors and limited access to healthcare resources are particularly pronounced in developing countries. Consequently, a large proportion of cataracts worldwide remain untreated. As the world's largest developing country, China, with a population of 1.4 billion in 2019, faces significant challenges. The main unique challenges facing China are the acceleration of population aging and the uneven allocation of urban and rural medical resources. Compared with other developing countries, China is aging faster, which leads to a rapid increase in the number of cataracts. In addition, insufficient medical resources and weak health awareness in rural areas have led to a low coverage of early diagnosis and treatment of cataracts. Although the government has made efforts to improve quality of life nationwide, China continues to struggle with population growth and disparities in income and access to healthcare services. Disease burden, cataract surgery rates/coverage, and human resources have been recognized by the World Health Assembly as key indicators for monitoring eye care services (15). The health burden of disease can be quantified using years lived with disability (YLDs) (16). To better assess the burden of cataracts in developing countries, we used the prevalence and YLD data from the Global Burden of Disease (GBD) 2021 study as key metrics to compare cataract patients in China by year, age, region, and gender. Our goal is to raise public awareness of cataracts in developing countries, promote timely diagnosis and treatment, and provide insights for the formulation of healthcare policies in these regions.

## Methods

### Data extraction

The research data is sourced from the GBD 2021 study (https://vizhub.healthdata.org/gbd-results/). The raw data in the GBD study comes from population censuses, household surveys, civil registries, vital statistics, disease registries, and health service utilization data. Epidemiological indicators for 369 diseases and injuries across 204 countries and regions, including incidence, prevalence, mortality rates, years of life lost (YLLs), years lived with disability (YLDs), and disability-adjusted life years (DALYs), are estimated. The uncertainty intervals (UI) for each indicator are generated using the 25th and 975th ordered 1,000 posterior distribution values (16).

This study collected data from the GBD 2021 study on the number of prevalence, crude prevalence rate (CPR), and age-standardized prevalence rate (ASPR), as well as YLDs number, YLD rates, and age-standardized YLD rates for cataract-related blindness and vision impairment in China, neighboring countries, and GBD super regions from 1990 to 2021. The CPR is reported as the number of cases per 100,000 population, while the ASPR is further adjusted for age structure. According to the WHO classification of vision impairment: moderate visual impairment is defined as presenting visual acuity (PVA) ≥6/60 and < 6/18, severe visual impairment as PVA ≥3/60 and < 6/60, and blindness as PVA < 3/60 or a central visual field radius < 10° (17).

### Joinpoint regression analysis

The joinpoint regression program 5.2.0 software was used to analyze the time trends of cataract-related blindness and vision impairment prevalence, as well as YLDs, in China from 1990 to 2021. The joinpoint regression analysis uses segmented regression within a logarithmic linear model to identify trend inflection points. The grid search method (GSM) calculates all potential join points, selecting the one with the smallest mean squared error (MSE) as the optimal inflection point. The number of optimal joinpoints was determined using Monte Carlo permutation tests (18). The final model calculated the annual percentage change (APC) and average annual percentage change (AAPC). The calculation formula for APC is:


$$\begin{array}{l} APC=\left(e^{\beta }-1\right)*100\% \end{array}$$

where β is the regression coefficient from the log-linear model ln(*y*) = β^*^x+*constant*. AAPC reflects the overall trend change by weighting each segment's APC according to the time span.

### Decomposition analysis

Through decomposition analysis, the contributions of aging, population growth, and epidemiological changes to the variations in cataract-related blindness and vision impairment prevalence and YLDs were quantified (19). The impact of each factor on the overall trend was calculated, providing a clearer understanding of the driving factors behind the increase in blindness and vision impairment. Specifically, we calculated YLDs for using the following formula:


$$\begin{array}{l} YLD_{a_{y},p_{y},e_{y}}=\overset{20}{\underset{i=1}{\sum }}\left(a_{i,y}*p_{y}*e_{i,y}\right) \end{array}$$

Where *YLD*_*a*_*y*_, *p*_*y*_, *e*_*y*__ represents the YLDs based on the age structure, population size, and YLDs rate for a specific year *y*; *a*_*i, y*_ represents the proportion of the population in age group *i* out of 20 age groups in year *y*; *p*_*y*_ represents the total population in year *y*; and *e*_*i, y*_ represents the YLDs rate in age group *i* in year *y*.

### ARIMA model

The ARIMA model (Autoregressive Integrated Moving Average Model) is a commonly used time series analysis method. We applied the ARIMA model to predict the CPR and crude YLD rates of cataract-related blindness and vision impairment from 2022 to 2035. The ARIMA model effectively captures trends and seasonal variations in time series data by combining three components: autoregression (AR), differencing (I), and moving average (MA). Auto. arima function was used to select the best optimization model based on Akaike information criteria, and the Ljung Box test was used to check whether the residual sequence was white noise. In addition, we performed stratified comparisons by gender and severity of vision injury.

### Statistical analyses

Data were organized and analyzed using R 4.4.1. The prevalence and YLDs of cataract-related blindness and vision impairment in China, surrounding countries, and the GBD super regions from 1990 to 2021 were analyzed. All analyses employed appropriate statistical models, including joinpoint regression analysis, decomposition analysis, and the ARIMA model. A *p*-value of < 0.05 was considered statistically significant.

## Results

**Figure 1:**
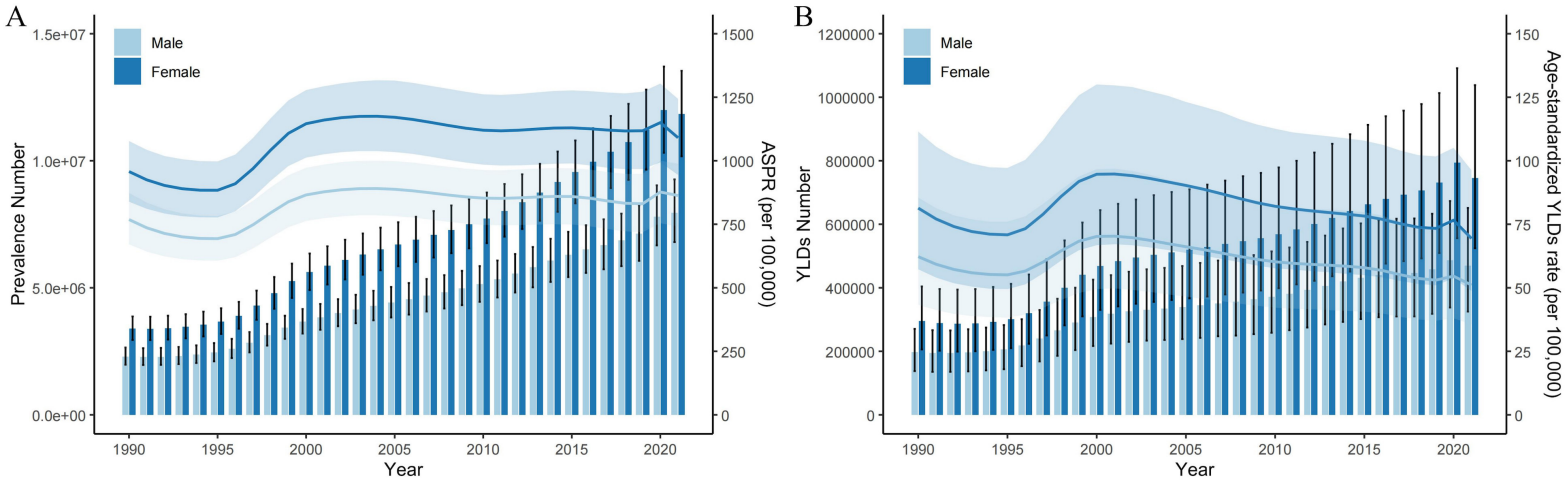
Prevalence and YLDs of cataract related blindness and visual impairment from 1900 to 2021 in China. Prevalence **(A)**, YLDs **(B)**. Error bars indicate the 95% uncertainty interval (UI) for the number of cases. Shading indicates the 95% UI for the rates. ASPR, age-standardized prevalence rate; YLDs, years lived with disability.

From 1990 to 2020, the number of prevalence and YLDs associated with cataract-related blindness and vision impairment in the Chinese population showed an upward trend, peaking in 2020 before declining. The age-standardized prevalence rate of blindness and vision impairment remained relatively stable after 2000, while the age-standardized YLD rate gradually decreased, with a single increase observed in 2020. The prevalence and YLDs of cataract-related blindness and vision impairment were higher in females than in males, as shown in Figure 1.

In 2021, the prevalence of cataract-related blindness and vision impairment in the Chinese population was 19,784,672 cases, with a CPR of 1,390.60 per 100,000 and an ASPR of 989.58 per 100,000. Compared to 1990, the AAPC was 4.20%, 3.52%, and 0.47%, respectively. The YLDs number for cataract-related blindness and vision impairment in 2021 was 1,215,072, with a crude YLD rate of 85.40 per 100,000 and an age-standardized YLD rate of 61.39 per 100,000. Compared to 1990, the AAPC for YLDs was 3.15%, 2.52%, and −0.44%, respectively. The gender-specific changes are presented in Table 1.

**Table 1:** Prevalence and YLDs of cataract-related blindness and vision impairment in China in 1990 and 2021.

**Year**	**Prevalence**			**YLDs**		
	**Number (95% UI)**	**Crude rate**^a^ **(95%UI)**	**Age-standardized rate**^a^ **(95%UI)**	**Number (95%UI)**	**Crude rate**^a^ **(95%UI)**	**Age-standardized rate**^a^ **(95%UI)**
**Both**						
1990	5,683,932 (4,917,940 to 6,513,736)	483.14 (418.03 to 553.67)	878.30 (770.53 to 991.83)	492,410 (342,706 to 674,502)	41.86 (29.13 to 57.33)	73.40 (51.60 to 100.62)
2021	19,784,672 (16,950,247 to 22,757,901)	1,390.60 (1,191.38 to 1,599.58)	989.58 (853.64 to 1,123.44)	1,215,072 (851,013 to 1,686,733)	85.40 (59.81 to 118.55)	61.39 (42.97 to 85.13)
AAPC	4.20 (4.01,4.38)	3.52 (3.06 to 3.98)	0.47 (0.22 to 0.72)	3.15 (2.849,3.451)	2.52 (2.21 to 2.82)	−0.44 (−0.70 to −0.18)
**Male**						
1990	2,290,328 (1,972,850 to 2,649,083)	377.42 (325.10 to 436.54)	768.50 (671.37 to 872.85)	197,290 (137,432 to 270,528)	32.51 (22.65 to 44.58)	62.25 (43.33 to 85.52)
2021	79,49,200 (6,796,812 to 9,265,885)	1,091.77 (933.50 to 1,272.60)	863.08 (740.74 to 987.69)	469,181 (325,131 to 651,501)	64.44 (44.65 to 89.48)	51.08 (35.70 to 70.67)
AAPC	4.08 (3.51 to 4.67)	3.54 (3.12 to 3.95)	0.43 (0.00 to 0.87)	2.95 (2.64 to 3.26)	2.35 (2.01 to 2.70)	−0.52 (−0.75 to −0.28)
**Female**						
1990	3,393,604 (2,946,900 to 3,871,411)	595.78 (517.35 to 679.65)	957.40 (838.66 to 1,077.58)	295,119 (205,858 to 404,284)	51.81 (36.14 to 70.97)	81.37 (57.29 to 111.48)
2021	11,835,471 (10,175,028 to 13,543,542)	1,703.82 (1,464.79 to 1,949.71)	1,090.60 (944.10 to 1,243.31)	745,891 (524,477 to 1,038,109)	107.38 (75.50 to 149.44)	69.41 (48.75 to 96.31)
AAPC	4.25 (4.04 to 4.45)	3.58 (3.37 to 3.78)	0.55 (0.29 to 0.82)	3.24 (2.91 to 3.56)	2.58 (2.26 to 2.90)	−0.356 (−0.64 to −0.08)

^a^per 100,000 population.

UI, uncertainty interval; AAPC, average annual percent change; YLDs, years lived with disability.

**Figure 2:**
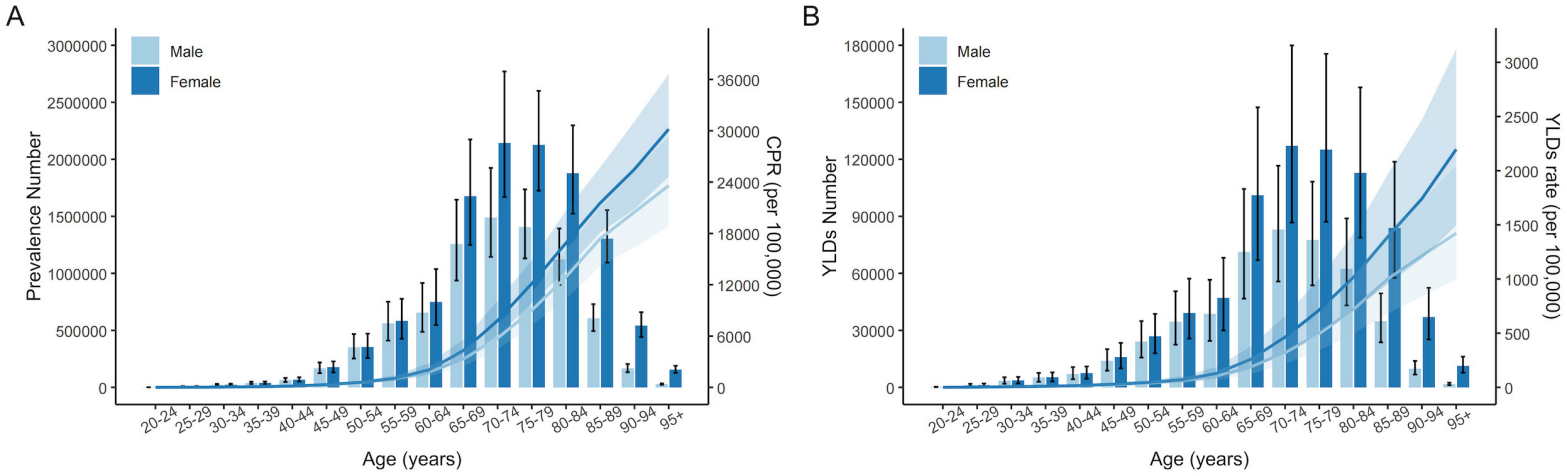
Age-specific prevalence and YLDs attributable to cataract-related blindness and vision impairment by sex in 2021. Prevalence **(A)**, YLDs **(B)**. Error bars indicate the 95% uncertainty interval (UI) for the number of cases. Shading indicates the 95% UI for the rates. CPR, crude prevalence rate; YLDs, years lived with disability.

As age increases, the number of prevalence and YLDs associated with cataract-related blindness and vision impairment first rise and then decline, peaking in the 70–74 age group. The majority of cases and YLDs are concentrated in the 65–89 age range. Both CPR and crude YLD rates consistently increase with age, showing a more pronounced rise after 60 years. In all age groups, females have higher prevalence and YLDs than males. See Figure 2 for details.

**Figure 3:**
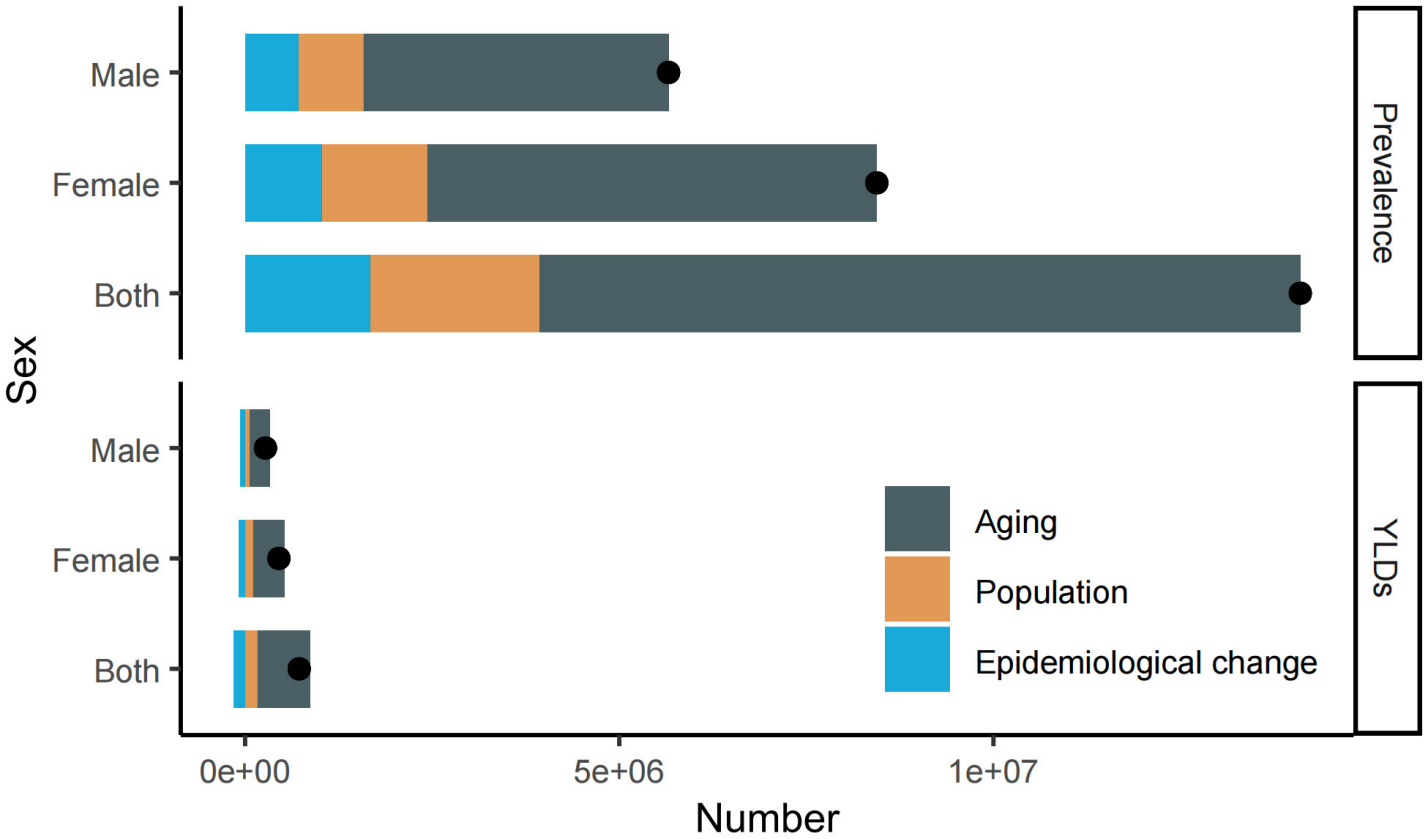
Population-level determinant changes in aging, population growth, and epidemiological changes for cataract related blindness and visual impairment in China by gender from 1990 to 2021. Black dots represent the total change contributed by all three components. A positive value for each component indicates a corresponding increase in the number of cases of blindness and VI, while a negative value indicates a corresponding decrease. YLDs, years lived with disability.

From 1990 to 2021, the number of prevalence and YLDs due to cataract-related blindness and vision impairment increased significantly. Aging contributed to 72.12% of the growth in cases and 98.3% of the increase in YLDs, while population growth accounted for 16.06% and 22.19%, respectively. Epidemiological factors contributed 11.81% to the rise in cases but had a negative impact on YLDs, with a decrease of −20.49%. The demographic factors by gender are shown in Table 2 and Figure 3.

**Table 2:** Changes in number of cases and YLDs of cataract according to population-level determinants from 1990 to 2021.

**Gender**	**Overall difference**	**Aging**	**Population**	**Epidemiolog-ical change**
**Prevalence**				
Male	5,658,872.12	4,079,110.18 (72.08%)	873,580.83 (15.44%)	706,181.1 (12.48%)
Female	8,441,867.08	6,009,004.14 (71.18%)	1,410,667.47 (16.71%)	1,022,195.46 (12.11%)
Both	14,100,739.19	10,170,139.85 (72.12%)	2,265,135.7 (16.06%)	1,665,463.64 (11.81%)
**YLDs**				
Male	271,890.6	273,722.35 (100.67%)	60,245.22 (22.16%)	−6,2076.97 (−22.83%)
Female	450,771.98	429,737.1 (95.33%)	101,559.04 (22.53%)	−80,524.16 (−17.86%)
Both	722,662.58	710,341.51 (98.3%)	160,375.46 (22.19%)	−148,054.39 (−20.49%)

YLDs, years lived with disability.

**Figure 4:**
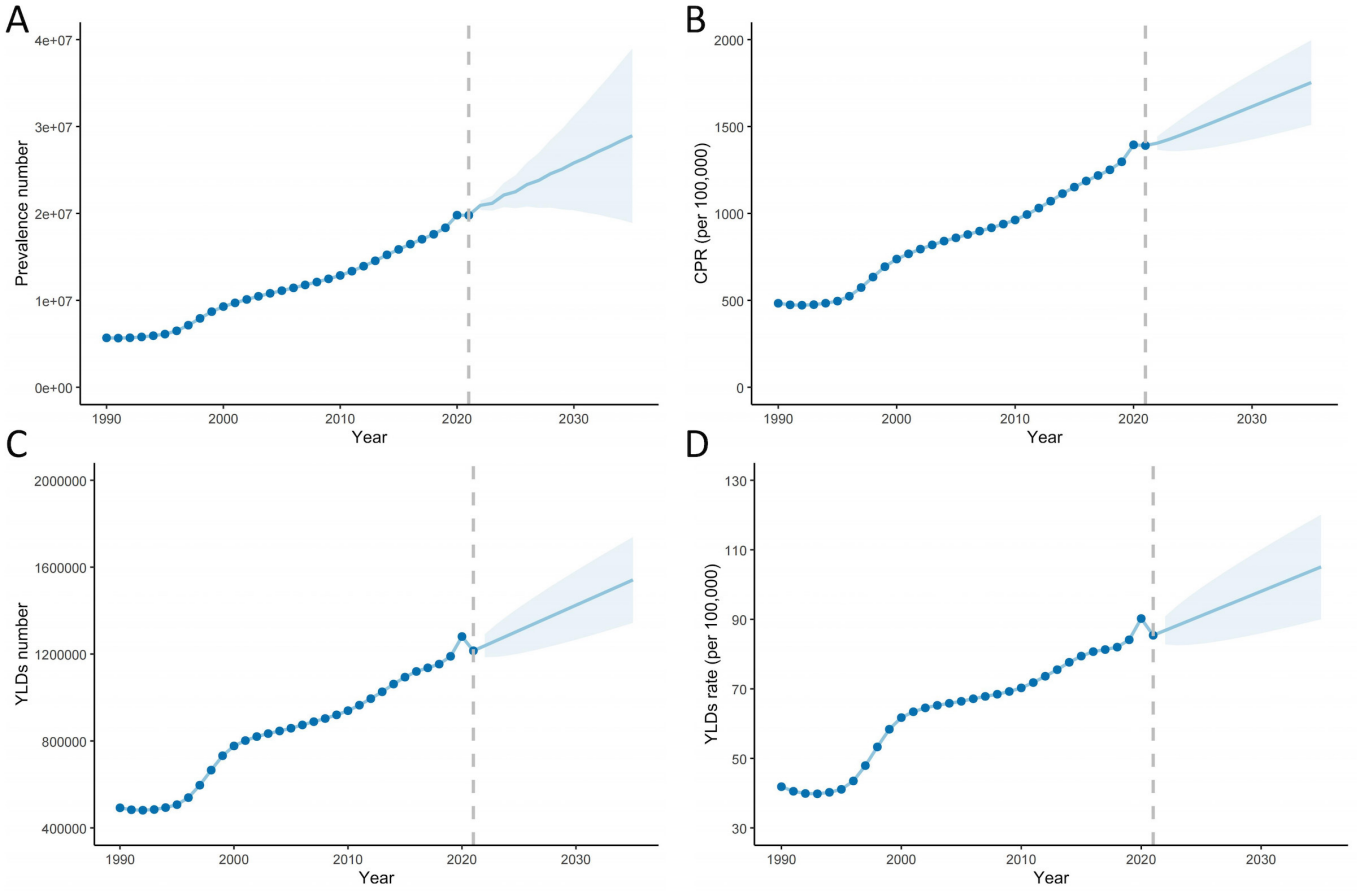
Predicted trends of prevalence and YLDs of cataract related blindness and visual impairment in China over the next 14 years (2022–2035). Prevalence number **(A)**, CPR **(B)**, YLDs number **(C)**, crude YLD rates **(D)**. CPR, crude prevalence rate; YLDs, years lived with disability.

Our study applied the ARIMA model to predict the number of prevalence, CPR, YLDs number, and crude YLD rates due to cataract-related blindness and vision impairment from 2022 to 2035. The optimized model selections were (1,2,0) for cataract-related blindness and vision impairment prevalence, (1,1,0) for CPR, (0,1,0) for YLDs, and (0,1,0) for the crude YLDs rate, with AIC values of 840.6, 277.24, 703.44, and 135.43, respectively, as determined by the auto.arima() function. The Ljung-Box test confirmed that the model residuals exhibited white noise (χ^2^ = 1.5435, *P* = 0.9988; χ^2^ = 2.4313, *P* = 0.9918; χ^2^ = 9.13, *P* = 0.5198; χ^2^ = 13.735, *P* = 0.1854). The predicted trends are shown in Figure 4. From 2022 to 2035, the prevalence and YLDs related to cataract are expected to increase. By 2035, the number of cataract-related cases and YLDs will reach 28,938,457 and 1,541,436, respectively. The CPR and crude YLD rates will be 1,753.00/100,000 and 105.07/100,000, representing increases of 26.06% and 23.03%, respectively, compared to 2021.

**Figure 5:**
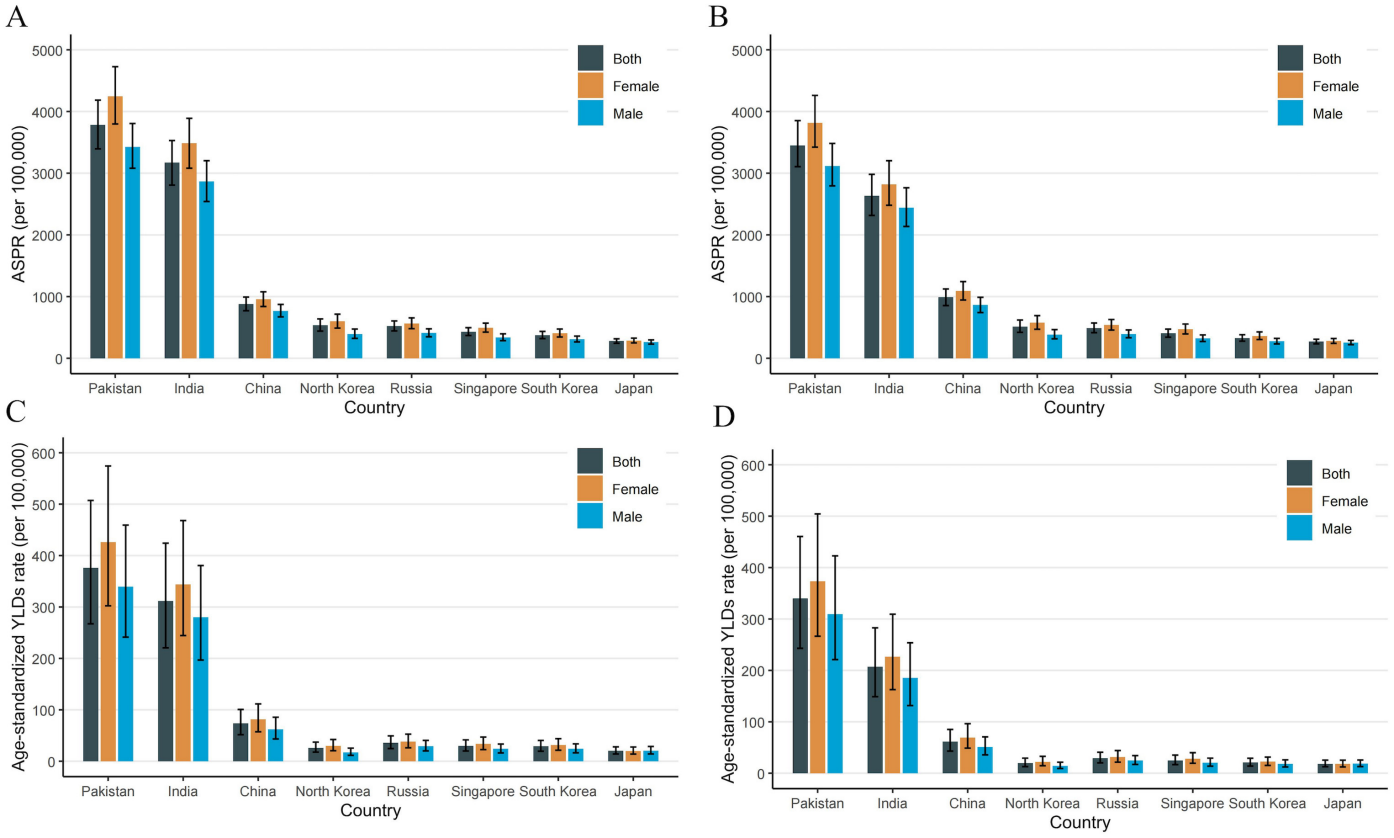
Distribution of ASPR and age-standardized YLDs rate due to cataract related blindness and visual impairment in China and other seven neighboring countries in 1990 and 2021. ASPR in 1990 **(A)**, ASPR in 2021 **(B)**, age-standardized YLDs rate in 1990 **(C)**, age-standardized YLDs rate in 2021 **(D)**. ASPR, age-standardized prevalence rate; YLDs, years lived with disability.

The ASPR and age-standardized YLD rates of cataract-related blindness and vision impairment in China and its neighboring countries in 1990 and 2021 are shown in Figure 5. In both 1990 and 2021, China ranked third in terms of ASPR and age-standardized YLD rates, with Pakistan and India ranking first and second, respectively. Japan had the lowest ASPR and age-standardized YLD rates. In all countries, the ASPR and age-standardized YLD rates for females were higher than those for males.

**Figure 6:**
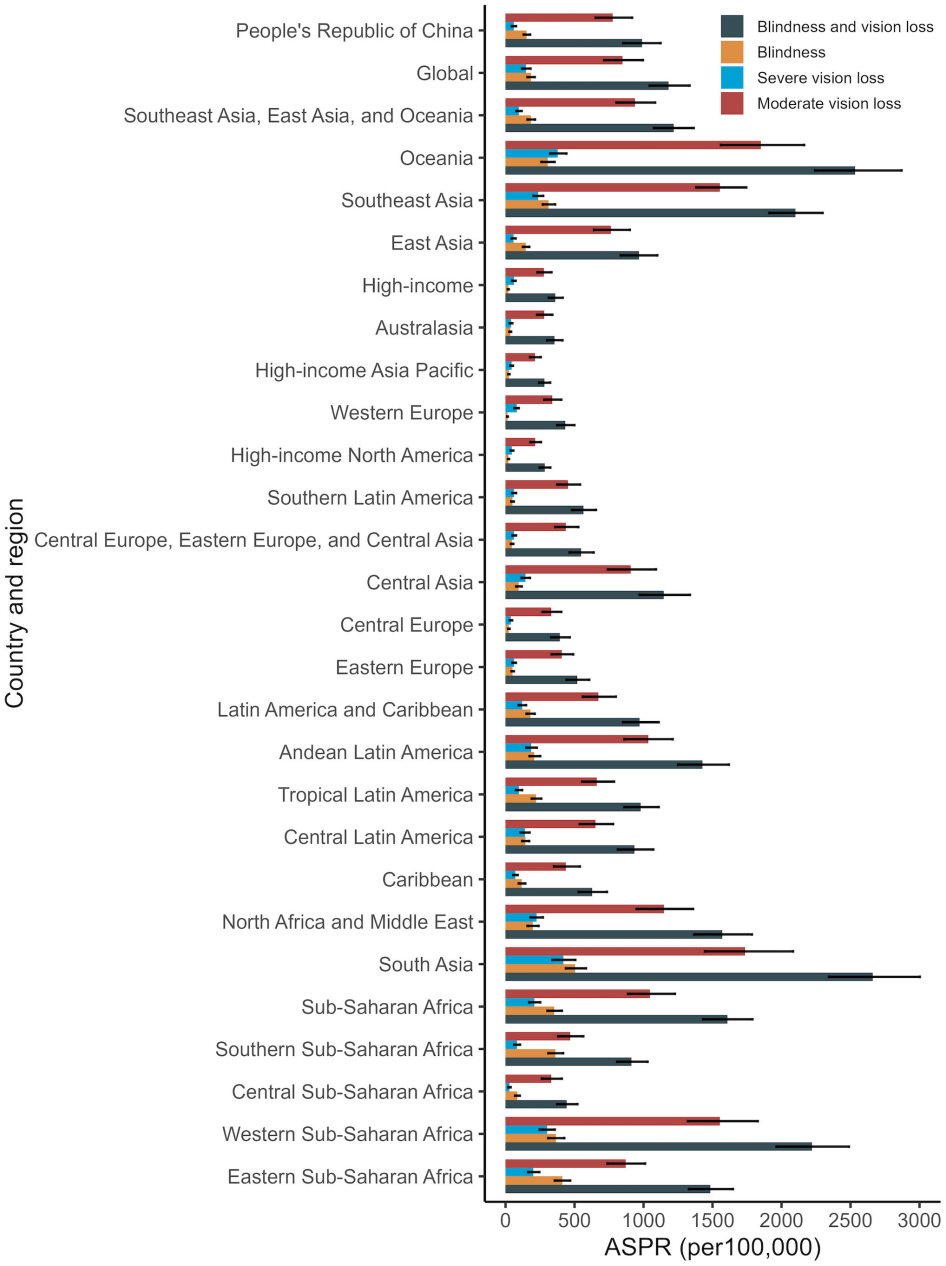
ASPR of cataract related blindness, moderate, severe and all vision impairment by GBD super regions in 2021. ASPR, age-standardized prevalence rate.

**Figure 7:**
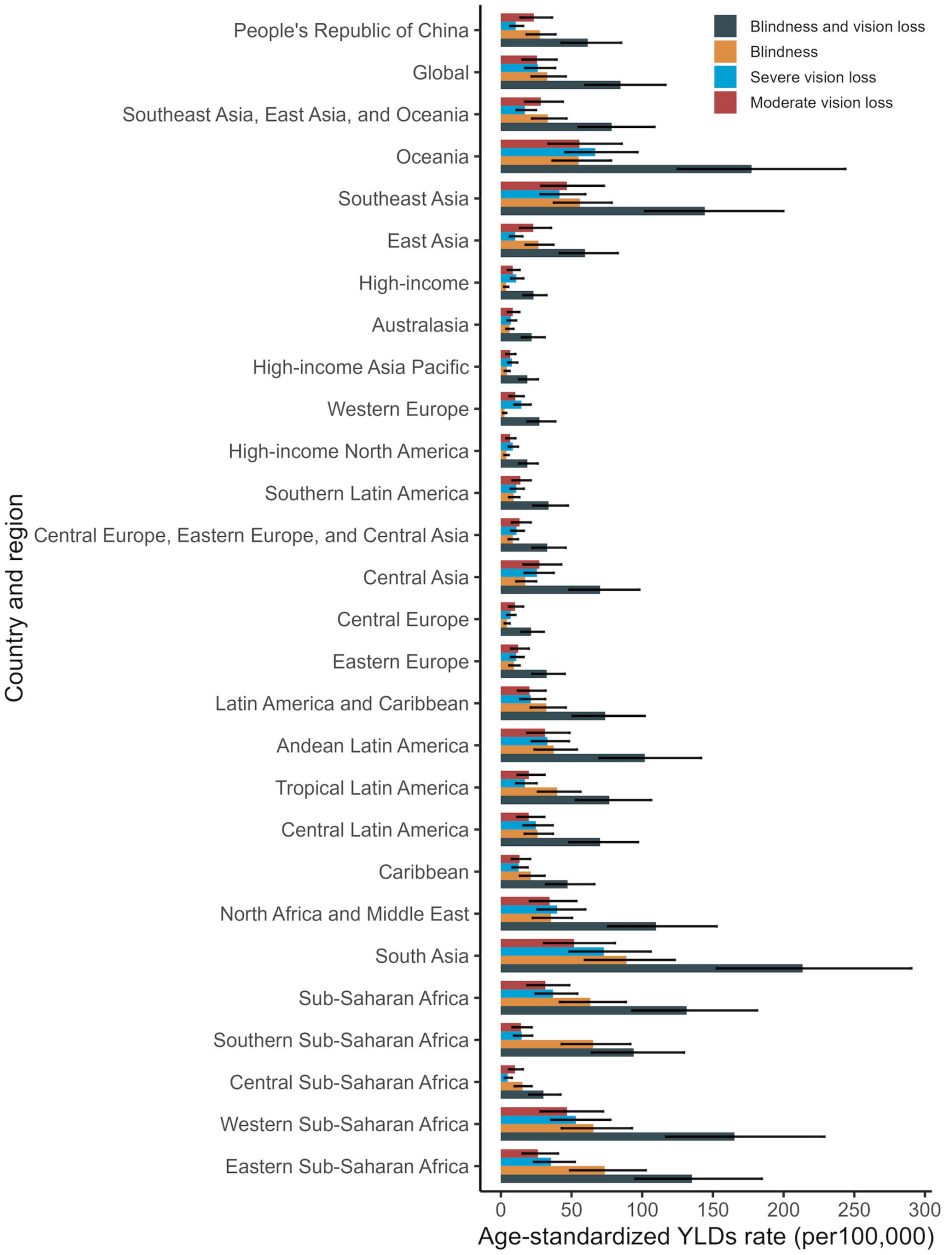
Age-standardized YLDs rate of cataract related blindness, moderate, severe and all vision impairment by GBD super regions in 2021. YLDs, years lived with disability.

In 2021, the ASPR and age-standardized YLD rates for cataract-related blindness and vision impairment in China and the seven GBD super regions are shown in Figures 6, 7, respectively. In China, the ASPR for cataract-related moderate vision impairment, severe vision impairment, and blindness were 776.48 per 100,000, 60.08 per 100,000, and 153.02 per 100,000, respectively. The corresponding age-standardized YLD rates were 23.34 per 100,000, 10.56 per 100,000, and 27.51 per 100,000, all lower than the global averages. Globally, high-income regions experienced the lowest disease burden from cataract-related blindness and vision impairment, while South Asia had the highest.

## Discussion

Although some studies have assessed the health and economic burden of cataract globally, there is limited research on the burden of cataract in developing countries, particularly in China, which has a large population. This study comprehensively examines the burden of cataract-related blindness and visual impairment by analyzing prevalence and YLD rates stratified by year, age, and gender. Additionally, we employ the ARIMA model to forecast the prevalence of cataract-related blindness and visual impairment in China by 2035. Our findings reveal that gender inequality in the burden of cataract has persisted in China since 1990. The prevalence of cataract-related blindness and visual impairment increases with age, showing a significant rise after the age of 60 and peaking in the 70–74 age group. Between 1990 and 2021, the age-standardized prevalence of cataract-related blindness and visual impairment exhibited a slight overall upward trend, while the age-standardized YLD rate remained relatively stable, with a gradual decline observed after 2000. By eliminating the influence of population size and age structure, age-standardized rates reflect the true burden of the disease. These results suggest that China has made progress in cataract diagnosis and treatment, likely due to the increasing implementation of community-based eye disease screening programs. In addition, the Chinese government has implemented policies providing free or subsidized cataract surgeries for impoverished patients, which has significantly enhanced the accessibility and coverage of cataract surgeries. However, given the challenges posed by population growth and aging, substantial efforts are still required to mitigate the visual impairment caused by cataracts.

Our study found that gender inequality in the burden of cataract has persisted since 1990, with disparities in visual impairment observed across different age groups and countries. This finding aligns with results reported in previous studies (20). Interestingly, similar gender differences have been noted not only in cataract but also in uncorrected refractive error (21), age-related macular degeneration (22, 23), and diabetic retinopathy (24). One possible explanation for this disparity is women's longer life expectancy. According to data from the World Health Organization, women generally have a higher average life expectancy than men. For instance, statistics published by the National Bureau of Statistics of China indicate that in 2023, the average life expectancy for men was 73.64 years, while for women, it was 79.43 years (25). Another contributing factor could be the long-standing effects of gender inequality. Evidence suggests that women face disadvantages in education, employment opportunities, income distribution, and access to healthcare (26). Although 60% of cataract patients are women, men are 1.39 times more likely to undergo cataract surgery (27). Additionally, the progression of cataracts may be associated with estrogen levels (28, 29). Previous studies have shown that early menopause increases the risk of cataract development, possibly due to the antioxidant properties of estrogen. Estrogen may counteract the formation of transforming growth factor-beta, reduce oxidative stress in the lens, and thereby mitigate lens opacification (30). It is essential to raise health awareness among women and strengthen medical support for women, particularly those in rural areas.

We analyzed the influence of various demographic and epidemiological factors on the prevalence and YLDs for cataract-related blindness and visual impairment between 1999 and 2021 across genders. Our findings indicate that population aging has consistently been the dominant demographic factor driving the increasing burden of cataract. The growth of the total population also contributed to the prevalence of cataract-related blindness and visual impairment, although its impact was relatively smaller compared to population aging. Interestingly, the effect of epidemiological factors on YLD was negative. This could be attributed to advancements in medical technology, which have improved early diagnosis and treatment of cataract, thereby reducing the disease burden. Additionally, improved post-surgical visual outcomes may have minimized the impact of cataract on patients' quality of life. Epidemiological progress, such as early screening programs and more effective treatments, may have contributed to a decline in blindness rates. In the future, it is crucial to enhance multi-sector collaboration and health education to raise public awareness and preventive knowledge of cataracts. Additionally, early screening programs for cataracts should be further promoted in primary healthcare institutions to reduce the incidence of advanced-stage cataracts. When comparing China to seven neighboring countries, we found that China consistently ranked third in both the age-standardized prevalence rate (ASPR) and age-standardized YLD rate, with Pakistan and India occupying the top two positions. Compared to the global GBD super regions, ASPR and YLDs related to cataract blindness, and moderate to severe vision impairment in China are lower than the global averages. However, they remain significantly higher than those in economically developed regions such as North America and Europe. In contrast, the ASPR and YLDs in South Asia, Oceania, and Western Sub-Saharan Africa are significantly higher than the global averages. These findings suggest a negative correlation between cataract-related blindness and vision impairment with economic development. The burden of cataract-related blindness is notably higher in developing countries, and the disease burden is more concentrated in regions with lower socioeconomic status. Similar conclusions were drawn by Yan et al. based on the GBD 2017 data (31). Several patient-related factors contribute to the unsatisfactory surgical coverage rate in developing countries, such as the cost of surgery, lack of patient awareness, long distances to healthcare facilities, cultural beliefs, and fear (32–34). In addition, the uneven distribution of ophthalmologists, the lack of trained eye care professionals and necessary surgical equipment in primary healthcare institutions, and the increased time and economic costs associated with seeking medical care have collectively contributed to the low coverage rate of cataract surgeries.

Furthermore, using the ARIMA model, we predicted the number of cataract-related blindness and visual impairment cases in China by 2035. Our projections indicate that the number of blindness cases due to cataract will reach 28.93 million, representing a 26.06% increase compared to 2021. Similarly, the age-standardized YLDs are expected to reach 1.54 million, an increase of 23.03% from 2021. These findings suggest that severe visual impairment caused by cataract in China will continue to rise in the coming years. A recent study highlighted that the number of ophthalmologists worldwide varies with economic development, ranging from an average of 9 per million in developing countries to 79 per million in developed countries (35). This disparity underscores the urgent need to address the shortage of healthcare resources as the population continues to grow. In this context, leveraging digital technology, telemedicine, and artificial intelligence to assist in cataract screening and diagnosis becomes both feasible and necessary. In addition, promoting the implementation of internet hospitals can drive innovation and transformation in healthcare service models, effectively integrate hospital medical resources, and better meet patients' diagnostic and treatment needs. These technologies have the potential to fundamentally transform disease screening, diagnosis, and monitoring processes, enabling more accurate analysis of disease progression and facilitating improved and/or personalized treatment strategies. In summary, this study utilized the latest GBD database to evaluate the cataract burden in China, the largest developing country. The findings provide critical evidence for healthcare policymakers regarding the burden of cataract, emphasizing the need for universal, equitable, and sustainable healthcare services.

The limitations of this study cannot be ignored. First, the accuracy of the health burden estimates depends on the quality of the original data sources. In cases where data were unavailable, reliance on out-of-sample model predictions inevitably introduced some degree of bias. Second, the types of cataracts were not differentiated in the database, preventing the assessment of the burden associated with different cataract subtypes. Metabolic cataracts and complicated cataracts, which are secondary to systemic or ocular diseases, impose a greater disease burden compared to simple age-related cataracts. Additionally, our analysis was conducted solely at the national level and did not account for potential regional disparities in cataract burden across China, including possible heterogeneity between urban/rural areas and coastal/inland regions.

## Conclusion

Overall, while the age-standardized YLD rate for cataract in China has declined from 1990 to 2021, indicating some progress in cataract-related healthcare, the prevalence and YLD numbers for cataract-induced blindness and visual impairment have significantly increased, and gender disparities persist. By 2035, more than one million people in China are projected to be threatened by visual impairment caused by cataracts. There is an urgent need for improved prevention and treatment strategies, with a particular focus on enhancing ophthalmic care services for women. Developing countries must expand cataract services to address the growing burden of cataract-related vision loss worldwide.

## Data Availability

The raw data supporting the conclusions of this article will be made available by the authors, without undue reservation.
